# Complexity As Key to Designing Cognitive-Friendly Environments for Older People

**DOI:** 10.3389/fpsyg.2016.01329

**Published:** 2016-08-30

**Authors:** Marica Cassarino, Annalisa Setti

**Affiliations:** ^1^School of Applied Psychology, University College CorkCork, Ireland; ^2^The Irish Longitudinal Study on Aging, Trinity College Dublin, The University of DublinDublin, Ireland

**Keywords:** environmental complexity, cognition, perceptual load, usability, environmental preference, aging

## Abstract

The lived environment is the arena where our cognitive skills, preferences, and attitudes come together to determine our ability to interact with the world. The mechanisms through which lived environments can benefit cognitive health in older age are yet to be fully understood. The existing literature suggests that environments which are perceived as stimulating, usable and aesthetically appealing can improve or facilitate cognitive performance both in young and older age. Importantly, optimal stimulation for cognition seems to depend on experiencing sufficiently stimulating environments while not too challenging. Environmental complexity is an important contributor to determining whether an environment provides such an optimal stimulation. The present paper reviews a selection of studies which have explored complexity in relation to perceptual load, environmental preference and perceived usability to propose a framework which explores direct and indirect environmental influences on cognition, and to understand these influences in relation to aging processes. We identify ways to define complexity at different environmental scales, going from micro low-level perceptual features of scenes, to design qualities of proximal environments (e.g., streets, neighborhoods), to broad geographical areas (i.e., natural vs. urban environments). We propose that studying complexity at these different scales will provide new insight into the design of cognitive-friendly environments.

## Introduction

With aging, the experience we have of the environment is reshaped both by physical, sensory, and cognitive changes, and by modifications of the perceived affordances offered by the environment. At the same time, the environment, in terms of architecture and sensory/cognitive stimulation provided, also shapes cognition and can be more or less supportive of independent living in older age. Thus, one could envisage a virtuous circle whereby the environment can provide an optimal level of stimulation to the older individual, so that she/he can maintain independence and, in turn, experience the environment in a positive and supportive way. Conversely, an environment which does not offer optimal stimulation can be detrimental for cognitive aging, unsupportive, and, likely, less pleasant for older people, to the detriment of their quality of life. In this targeted review we propose that the concept of complexity can provide a route to studying interactions between aging individuals and their environment, starting from sensations and perception, and including the lived experience of older adults in the environment.

## Environmental Measures Linking Complexity to Cognitive Aging

Lived environments offer both opportunities and challenges for healthy living (Vlahov and Galea, 2002; Jackson, 2003; Galea et al., 2005; Boyko and Cooper, 2011; Corburn, 2015). The extensive evidence that person–environment interactions influence human behavior (Barker, 1968; Lawton and Nahemow, 1973; Bronfenbrenner, 1979; Bronfenbrenner and Ceci, 1994; Wahl et al., 2012), and that characteristics of the built environment contribute to physical and mental health (Badland and Schofield, 2005; Brennan Ramirez et al., 2006; Renalds et al., 2010; Kerr et al., 2012; Dallat et al., 2014), has urged to reconsider environmental planning and design as more user-centered (Gehl, 2010; Gehl and Svarre, 2013) and, in the light of global aging and urbanization (World Health Organization, 2007; Beard and Petitot, 2010), more facilitating for aging individuals, or “age-friendly” (World Health Organization, 2002, 2007, 2012). Understanding how lived environments are experienced by older people has received growing interest in research (Phillipson, 2011; Buffel et al., 2012), and given the crucial role of cognitive health in maintaining autonomy and quality of life in older age (World Health Organization, 2002), many studies have explored the beneficial influence of factors such as social activities and lifestyle on cognitive aging (Fillit et al., 2002; Hertzog et al., 2008; Stine-Morrow et al., 2008; Stern, 2009, 2012). However, only recently research has started to systematically address the influence of physical and perceptual characteristics of the environment on cognitive functioning in older age (Wu Y.-T. et al., 2014; Cassarino and Setti, 2015).

The present paper argues that trajectories of cognitive aging as well as day-to-day cognitive performance of older people can be affected by environmental factors which make places more or less complex for older people, and that environmental complexity could represent an important and measurable contributor to cognitive functioning (Rapoport and Kantor, 1967; Rapoport and Hawkes, 1970; Rapoport, 1990; Davidson and Bar-Yam, 2006). Effectively, environmental complexity could be a potentially measurable contributor to cognitive reserve (Stern, 2009): Animal studies have shown that exposure to enriched, complex environments, presenting elements of novelty, can have a direct impact on brain structure and cognition (Rosenzweig et al., 1972; Diamond, 2001; Cassarino and Setti, 2015). Enriched environments may also promote an active lifestyle, e.g., physical activity, which in turn is associated with better cognitive performance in older age (Cassarino and Setti, 2015).

The purpose of the present work is to explore links between cognitive aging and existing measures of environmental complexity by considering studies on perceptual stimulation, environmental preference, and perceived usability of lived environments at different environmental scales (Jackson, 2003; Kim et al., 2014; Cassarino and Setti, 2015), going from visual and/or auditory micro-characteristics of scenes (micro scale), to design qualities of streets and neighborhoods (meso scale), to broad forms of environmental exposure (macro scale: urban vs. natural).

**FIGURE 1:**
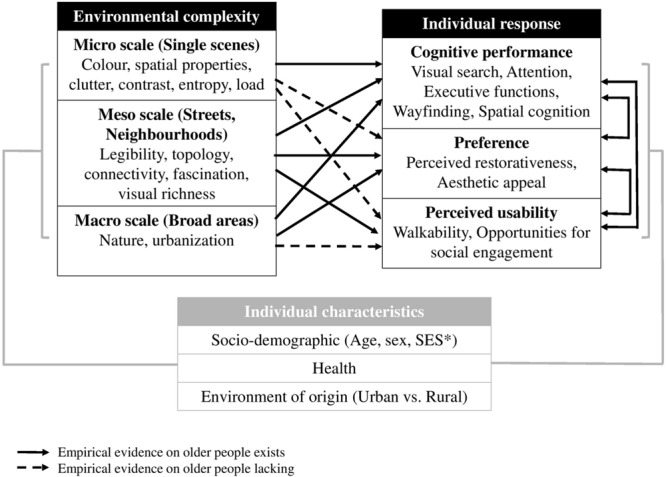
**Links between environmental complexity and cognition.** Proposed framework to study the association between environmental complexity (defined at multiple environmental scales) and cognitive performance in aging. Solid lines indicate established associations (e.g., environmental perceptual stimulation can be associated with cognitive performance in older age directly in relation to cognitive load). Dashed lines indicate associations related to aging processes which need to be explored by future research. Individual characteristics (in gray) mediate the association. ^∗^SES, socioeconomic status.

**Figure 1** synthesizes a framework based on measures of complexity which are directly or indirectly associated with cognitive health at different environmental scales, as well as the links between these measures. In the framework, some links have been already explored in the literature in relation to aging (indicated by solid lines in **Figure 1**), while other links (indicated by dashed lines in **Figure 1**) are suggested/inferred and need empirical exploration.

The framework is based on the assumption that cognition is situated (Clark, 1999a,b), embedded in the environment. The literature on learning environments (Brown et al., 1989; Choi and Hannafin, 1995) and ecological models of development (Bronfenbrenner, 1979; Gibson, 1986, 2000) suggest that the successful fulfillment of cognitive tasks depends on how individuals interact with their surroundings. This interaction can be explored in relation to three types of environmental influences:

(a) the direct environmental impact on cognitive functioning based on the amount/type of perceptual information (Lavie, 1995; Lavie et al., 2004; Berman et al., 2008; Linnell et al., 2013);
(b) the mediating role of environmental qualities which influence affective responses such as environmental preference (Kaplan and Kaplan, 1989; Kaplan, 1995), as well as
(c) the “affordances” or “presses” which affect the perception of usability and, as a consequence, the likelihood of using the environment (Lawton and Nahemow, 1973; Gibson, 1986).

We argue that defining complexity in relation to these different dimensions may provide insights into studying the environmental impact on cognitive aging, especially considering that the evidence for the impact of these dimensions on cognition is abundant.

The plausibility of a direct environmental impact on cognition has been supported by animal studies (Engineer et al., 2004; Herring et al., 2009; Hannan, 2014), as well as recent epidemiological evidence on geographical variations of cognitive functioning in aging when socio-economic and lifestyle factors were controlled for (Wu et al., 2015; Cassarino et al., 2016). Experimental evidence on environmental restorativeness for cognitive skills, i.e., the potential for natural, green environments to restore depleted attentional capacities as described within attention restoration theory (Kaplan and Kaplan, 1982; Kaplan, 1995; Hartig et al., 2003; Berman et al., 2008), also suggests a direct link between environment and cognition in older adults (Gamble et al., 2014). Specifically, ART suggests that exposure to nature helps to restore humans from attentional fatigue and stress (Berto, 2014) due to the presence of perceptual stimulation that engages bottom-up attention (or involuntary attention) without causing a burden on top-down attentional resources (defined as directed or voluntary attention) which can be used for other cognitive tasks, such as for example successfully navigating a novel environment. This hypothesis has recently received support from neuroimaging studies showing that exposure to environments with high restorative potential, such as natural scenes, or urban scenes including vegetation, activate brain areas involved in involuntary attention (Martínez-Soto et al., 2013), including the middle frontal gyrus, middle and inferior temporal gyrus, insula, inferior parietal lobe, and cuneus.

User’s environmental preference can further inform on environmental influences on cognition because it is related to how, and based on which factors, people perceive the surrounding environment as pleasant (Lynch, 1960; Quercia et al., 2014; Zambaldi et al., 2014). Studies on environmental restorativeness have in fact shown that cognitive skills such as voluntary attention and executive functions are positively associated with preference ratings of lived environments (Kaplan and Kaplan, 1982; Kaplan and Berman, 2010). Moreover, the aesthetic appeal of the environment can influence lifestyle, such as transportation choices (Kerr et al., 2012; Ding et al., 2014).

Lastly, the design of the built environment influences its perceived usability, for example in terms of opportunities for physical exercise, and therefore the engagement in active lifestyles (Ewing and Handy, 2009; Guo, 2009; Carlson et al., 2012; Kerr et al., 2012), which in turn benefit cognitive health, especially in older age (Fillit et al., 2002; Abbott et al., 2004; Fratiglioni et al., 2004; Weuve et al., 2004; Ble et al., 2005; Erickson et al., 2011). For example, the successful navigation of an environment (e.g., a city) for an older individual depends not only on the person’s visuo-spatial skills, but also on the opportunities for navigation present in that environment (e.g., accessible pedestrian areas), and on the aesthetic appeal which promotes positive psychological states (e.g., presence of green (Berto, 2014).

The relationship between environmental complexity and the aging individual’s cognitive skills may influence whether the person is able to use the environment finding it easy to use, pleasant and conducive to an active lifestyle. In turn, such a positive relationship with the environment may promote healthy cognitive aging. Environmental complexity could represent a key factor to identify an optimal level of environmental stimulation for cognitive functioning in older age, however, it is difficult to provide a definition of complexity that could be studied in relation to all the above dimensions, and inform cognitive aging in relation to different types of environment. In fact, there is no commonly accepted operationalization of complexity in the literature (Cannon and St John, 2007), although recent studies have attempted to operationalize the construct (Berto et al., 2015).

Looking at micro features of scenes, for example, measures of visual complexity include (but are not limited to, see Cavalcante et al., 2014; Gunawardena et al., 2015, for a review): clutter, defined by Rosenholtz et al. (2005) as an excessive amount of distractors in a scene, determined either objectively through statistical techniques (Rosenholtz et al., 2012; Jingling and Tseng, 2013) or subjectively via participants judgments (Ho et al., 2001; McPhee et al., 2004; spatial frequency, defined as a measure of the repetition of sinusoidal components of a structure per unit of distance (Cavalcante et al., 2014); contrast, defined in vision as the difference in luminance or color that makes an object or display distinguishable from others (Rosenholtz et al., 2005; Cavalcante et al., 2014); fractal dimension (a measure of how well an object fills the space in which it lies, the higher the fractal dimension the higher the visual complexity, Mandelbrot, 1977).

Moving onto the meso scale of qualities of the built environment, complexity has been measured in terms of richness and variety of information in urban design (Kaplan et al., 1989; Ewing and Handy, 2009), while studies on space syntax use network connectivity as a measure of layout complexity (Slone et al., 2016).

Moreover, macro scale environments such as cities tend to be considered in research as more perceptually complex than rural and/or natural settings (Kaplan and Kaplan, 1989; Berman et al., 2008; Linnell et al., 2013, 2014).

These different measures are due to the specific characteristics of each field of investigation. However, numerous definitions of complexity make it difficult to operationalize this construct for a broad empirical examination of the environmental influence on cognition, justifying the need for a framework which synthesizes different measures of complexity to identify the links between cognition and the environment. This would allow to explore whether environmental complexity is associated with cognitive performance, preference and usability at each environmental scale (micro, meso, and macro), or whether the association at one scale may impact the association at other scale.

To this end, we discuss in the following sections a selection of studies on specific measures of complexity associated with cognitive performance, environmental preference, and perceived usability for each environmental scale as described in **Figure 1**. Although aging individuals are the population of interest of the present review, little research in this area has been carried on older people, therefore inferences on implications for studying cognitive aging are proposed where evidence on young populations is the only available. We then discuss suggestions for future research.

## Complexity and Cognitive Performance

At a micro scale, the association between complexity and cognitive performance has been investigated in terms of low-level perceptual features of images which influence visual search, showing, for example, that scenes high in complexity in terms of clutter (measured either objectively or subjectively) or crowding of distractors, impact negatively on reaction times and accuracy when trying to detect a target stimulus (Ho et al., 2001; Plainis and Murray, 2002; McPhee et al., 2004; Rosenholtz et al., 2005; Jingling and Tseng, 2013). These results may depend on the fact that visual complexity affects scanning strategies, as shown by Wu D.W.-L. et al. (2014) whom, by examining temporal dynamics of eye movements, reported less structured, and therefore more exploratory, scanning strategies for scenes with high complexity (measured in terms of fractal dimension and clutter) in young participants, while reduced complexity was associated with more structured fixations around specific objects. Davidson and Bar-Yam (2006) reported, however, positive associations between visual complexity, operationalized as a combination of possible spatial positions (a measure of entropy) and internal features of objects, and the cognitive performance of older adults measured through Mini Mental State Examination (MMSE). One might then ask whether there is a linear association between increased visual complexity and worse perceptual and voluntary attentional processing. Neurophysiological studies (Hansen et al., 2012) have shown that, in young adults, an increase in visual complexity actually stimulates enhanced responses by the visual system (measured through evoked potentials), but up to a certain threshold after which saturation is reached, supporting a detrimental effect on visual search for scenes which are perceptually too complex (Hansen et al., 2012; Cavalcante et al., 2014).

According to load theory (Lavie and Tsal, 1994; Lavie, 1995; Lavie et al., 2004), susceptibility to distractors depends on the level of perceptual load caused by an attended scene: higher perceptual load, associated with higher complexity, for example number of objects or colors, reduces the awareness for distractors. While this reduced distractibility indicates improved selective attention, it also implies lower visual and auditory awareness of stimuli which could be important in real-life situations, as for example the presence of unexpected events while driving (Murphy and Greene, 2015). Given age-related changes in visual processing (Sokol et al., 1981; Porciatti et al., 1992; Tobimatsu et al., 1993; Fiorentini et al., 1996), one could expect an even higher dependence of the visual system on visual complexity with aging. In fact, older age exacerbates the interference effects associated with visual complexity, as found for example in studies on simulated driving in different conditions of clutter or contrast (Ho et al., 2001; McPhee et al., 2004; Cantin et al., 2009), and is associated with higher susceptibility to distractors (Maylor and Lavie, 1998; de Fockert et al., 2009), meaning that low-level perceptual features which make the environment less complex could facilitate its successful exploration or navigation for an older person.

Considering complexity at the meso scale of global qualities of proximal environments (e.g., streets, neighborhoods), fascination (Kaplan, 1995; Kaplan and Berman, 2010) is a subjective quality of environments proposed by ART to elicit involuntary attention and therefore reduce the burden on directed (voluntary) attention, improving selective attention, for example measured through an attention orienting task (Berto et al., 2010), as well as promoting a less effortful visual search measured via eye movements (Berto et al., 2008). In addition, topographic factors are relevant to understand the burden of the structure of the environment on cognition, given the evidence that navigational skills can decrease with age (Lövdén et al., 2005; Liu et al., 2011; Klencklen et al., 2012). Legibility, defined by Lynch (1960) as the extent to which a place can be easily read to be navigated, has been shown to affect wayfinding in outdoor environments both in healthy individuals (Long and Baran, 2012; Li and Klippel, 2014), and in patients with dementia and cognitive impairment, for example in relation to the presence of landmarks and architectural features (Mitchell et al., 2004; Mitchell and Burton, 2006). Moreover, complex topology has been associated with reduced visual sampling in older patients with Parkinson’s Disease, when navigating environments with turning points rather than straight paths (Galna et al., 2012). In line with this evidence, Barton et al. (2014) found impaired navigation skills (measured in terms of speed and accuracy in reaching a target) in environments with low intelligibility, which they operationalized as the correlation of connectivity (the number of potential routes connected to a specific path in a network) and integration (the average number of turns required to change path in the network). The results were independent of familiarity with the environment or accessibility to visual information. Similarly, Slone et al. (2015) compared the wayfinding performance of young participants in two virtual indoor environments, by manipulating plan complexity, a measure of network connectivity defined as the average number of connections at each decision point or terminal corridor, and found that the more interconnected (more complex) environment caused more errors and longer completion times to reach a target, although performance improved with familiarity. In a following study (Slone et al., 2016) using functional magnetic resonance imaging (fMRI) the authors found that varying the network connectivity (and thus the complexity) of an environment not only influenced navigational performance, but also modulated the activity of brain areas associated with successful navigation (e.g., hippocampus, precuneus, cerebellum, and prefrontal cortex). Thus, legibility and topology are distinct but both associated with environmental complexity, and, importantly, with cognitive performance in terms of navigation skills.

Lastly, at a macro scale, different studies based on ART have reported the cognitive benefits of exposure to green (both for real environments and pictures) in young and older people, in terms of visual search (Sandry et al., 2012), as well as voluntary attention and executive functions (Laumann et al., 2001; Hartig et al., 2003; Berto, 2005; Berman et al., 2008; Kaplan and Berman, 2010; Berry et al., 2014; Gamble et al., 2014). Berman et al. (2012) also found improvements in memory span after a walk in nature for patients with depressive disorders. If a short exposure to urban or natural environments affects cognition, one might argue that different perceptual and top-down attentional strategies could be influenced by the environment of residence, which could therefore be considered as a form of long-term exposure. Studies which compared perceptual biases and attentional engagement of individuals living in remote rural areas to a highly urbanized group (de Fockert et al., 2011; Caparos et al., 2012; Linnell et al., 2013, 2014; Bremner et al., 2016) have shown that people living in urbanized areas (i.e., Londoners), when compared to remote individuals, had a more global perceptual bias and more unfocused selective attention, which would indicate more disengaged and exploratory visual strategies. The authors suggested that these differences were due to a higher level of visual clutter (in terms of number of objects) in urban environments, which would cause an increase in intrinsic alertness and would prioritize exploration over focused attention (Linnell et al., 2014). This effect, according to the authors, was independent of cultural or social influences because even a brief exposure (two visits) of remote people to an urbanized environment changed the perceptual bias (measured through susceptibility to the Ebbinghaus Illusion) from local to global (Caparos et al., 2012). In line with these results, Chapman and Underwood (1998) reported shorter fixations for drivers in urban rather than rural environments, suggesting more exploratory scanning strategies for complex environments. In our recent work (Cassarino et al., 2016), we showed that urban healthy older people had better executive functions than people living in rural areas after controlling for socio-economic, health, and lifestyle confounders, further indicating that different environments could be associated with distinct perceptual and cognitive abilities. Although the study did not manipulate environmental complexity directly, the results suggest a direct association between living in a complex environment and cognitive functioning in older age.

## Complexity and Environmental Preference

Low-level color and spatial properties of scenes have been associated with preference for environments which present elements of nature (Berman et al., 2014; Kardan et al., 2015). Specifically, Berman et al. (2014) showed that properties including lower density of straight edges, lower hue level (i.e., high prevalence of yellow–green content), and higher diversity in color saturation were more likely to be found in scenes of nature, and were significantly associated with positive ratings of environmental preference; the authors speculated that, in line with ART, these properties could explain preference for natural environments rather than urban scenes because less taxing on voluntary attentional resources. These results were replicated by Kardan et al. (2015), who showed that scenes of environments which presented varying edges, diverse levels of saturations, and yellow–green color tones significantly contributed to positive preference ratings in younger adults. Similarly, Quercia et al. (2014) reported positive aesthetic judgments of beauty, quiet and happiness for environmental scenes with green color, a higher density of vertical edges (a measure related to the structure of buildings), and a higher density of visual points of interest. In addition, Forsythe et al. (2011) showed that images of natural environments with high complexity, measured through fractal dimension, were judged as the most beautiful when compared to images of man-made environments as well as images of abstract art, and the objective complexity matched well with the subjective perception of complexity (defined in this study as “the amount of detail and intricacy”). However, despite the evidence that older people prefer natural environments (Berto, 2007), perceptual features of scenes associated with environmental preference have not been tested in older populations, thus representing an interesting area for future investigation. It is also to note that architectural micro features of urban streetscapes can influence environmental ratings, as found by Lindal and Hartig (2013) who associated higher architectural entropy, measured as variation in silhouette and surface attributes of buildings, with positive judgments of preference and likelihood of restoration, suggesting that different types of perceptual features can influence users’ appeal depending on the specific type of environment.

Studies on urban design (Rapoport and Kantor, 1967; Rapoport and Hawkes, 1970; Rapoport, 1990; Ewing et al., 2006; Ewing and Handy, 2009; Purciel et al., 2009) inform on perceived qualities associated with users’ environmental preference at a meso scale. Among other qualities, complexity defined as visual richness in colors, architectural styles, buildings and activities is a factor significantly influencing positive affective responses to places (Rapoport and Kantor, 1967; Ewing and Handy, 2009; Purciel et al., 2009). Similarly, Kaplan et al. (1989) hypothesized that complexity, defined as richness of environmental information, is a predictor of environmental preference because promoting exploration, and studies on the preference for urban landscapes seem to support Kaplan’s hypothesis, indicating natural elements as key modulator for positive ratings of urban environments (Herzog, 1992; Hernández and Hidalgo, 2005; Abkar et al., 2011; Pazhouhanfar et al., 2013; Martínez-Soto et al., 2014; Twedt et al., 2016). Along this line, richness and variety in environmental information has been suggested as key design factors for dementia-friendly environments (Mitchell and Burton, 2006).

More broadly, natural environments have been associated with positive judgments of preference (Herzog, 1992; Laumann et al., 2001; Hernández and Hidalgo, 2005; Abkar et al., 2011; Pazhouhanfar et al., 2013; Martínez-Soto et al., 2014; Twedt et al., 2016). A limitation of comparing broad environments such as green areas and urban contexts is the potential influence of confounders, which calls for a more in-depth analysis of these environments. A recent study (Staats et al., 2016) addressed this issue by comparing judgments of preference and restoration likelihood for four urban scenarios (city park, cafe, shopping mall, busy street): the results showed that busy street scenarios were the least preferred, although these results were moderated by social factors (being in company or alone). Interestingly, the findings were moderated by country of residence, which highlights the importance of broad contextual factors for environmental perception.

## Complexity and Perceived Usability

Gibson’s ecological theory of perception (Gibson, 1986) suggests that perceptual characteristics of the environment can act as “affordances” which inform users on opportunities for action, and which facilitate usability depending on how they fit individuals’ abilities. Importantly, environments that are perceived as usable have the potential to promote health-related behavior, such as physical activity, or walkability (Leyden, 2003; Cohen et al., 2007; Wood et al., 2008; McCormack et al., 2010; Adkins et al., 2012). Thus, identifying perceptual affordances in the environment can inform on strategies to foster active lifestyles which benefit cognitive health in older age. For example, street characteristics such as slopes or zebra crossings have been reported to be perceived by older people as more attractive for walking (Borst et al., 2008). Moreover, traffic lights can facilitate older people to cross the street, but if the lights do not allow enough time for older pedestrians to cross (Romero-Ortuno et al., 2010), they can negatively impact on mobility, especially if the older person finds it difficult to use perceptual information for decision-making (Lobjois and Cavallo, 2009). These features can be considered measures of complexity which inform on the accessibility of the environment for older people. However, while environmental measures to reduce complexity for enhanced usability have been to some extent implemented in studies on universal design in relation to accessibility for individuals with physical or cognitive impairment, for example in terms of street layout, (Mace, 1997; Mynatt et al., 2000; Iwarsson and Ståahl, 2003; Crews and Zavotka, 2006), an account linking low-level perceptual features with the experience and the use of the environment in normal aging is still lacking. One could expect that the same perceptual features of the environment that influence top-down attentional control and environmental preference, such as clutter or color properties, would affect its perceived usability, but to our knowledge no studies have explored this association, especially in relation to aging, which stimulates further research in this area, as suggested by Wu Y.-T. et al. (2014).

Complexity at a meso scale, defined as richness of information, can also promote the use of the environment (Rapoport and Hawkes, 1970; Ewing and Handy, 2009; Ewing et al., 2015). For example, in relation to walking, Ewing et al. (2015) found a significant positive association between the number of street furniture (an indicator of urban complexity in terms of visual richness) and the number of pedestrians encountered in a given block, although they didn’t record the age of the pedestrians. Nonetheless, studies on environmental design for physical activity in older people suggest that elements of attractiveness and interest increase perceived walkability (Michael et al., 2006; Kerr et al., 2012). On the other hand, however, perceptions of walkability are influenced by design qualities which make environments more accessible, such as legible topography or increased network connectivity (Guo, 2009; Adkins et al., 2012). These qualities have been in fact associated with positive perceptions of usability and walkability both in healthy older individuals (e.g., in relation to street connectivity and accessibility to services; see Rosso et al., 2011; Kerr et al., 2012), and in patients populations (Mitchell et al., 2004; Mitchell and Burton, 2006; Joseph and Zimring, 2007).

At a macro scale, in a previous review on environmental influences on aging processes (Cassarino and Setti, 2015), we compared urban and rural environments in relation to physical exercise and social engagement, showing how each type of environment was associated with both perceived opportunities and challenges for active and engaged lifestyles (e.g., some studies reported higher level of instrumental walking in rural areas, but more recreational walking in urban areas). Assuming that rural environments are less perceptually and structurally complex than urban contexts, and based on the evidence that environmental measures related to health-related behavior in aging can be area-specific (Cleland et al., 2015; Levasseur et al., 2015), one could argue that different environments afford different types of usability. While urban–rural dichotomies can be too simplistic to address usability, studies on nature highlight that the use of green areas (which are supposedly more available in rural environments) benefits physical and mental health (Barton and Pretty, 2010; Barton et al., 2011; Berman et al., 2012; Beyer et al., 2014; Dallat et al., 2014), in turn promoting cognitive health as well as restoring attention, as previously discussed.

## Discussion and Conclusion

The discussed literature indicates properties and qualities which make lived environments more or less complex, and how they may impact cognitive performance either directly or indirectly. Importantly, while measures of complexity have been discussed over three environmental scales (i.e., micro, meso, and macro), these need to be considered not as distinct, but as interconnected and interdependent levels of a continuum of environmental influences.

Considering different operationalizations of complexity at a micro scale, cognitive functioning in older age can be affected by properties that make scenes less perceptually complex, such as reduced clutter or presence of distractors, which have been shown to facilitate visual search and voluntary attention. Color and spatial properties which can be found in natural (and supposedly less complex) settings are more appealing to users, and ART suggests that environmental preference may depend on the restorative potential of nature for voluntary attention, drawing a link between affective and cognitive responses to the environment based on perceptual complexity which deserves further exploration in relation to aging. These properties could in fact potentially serve as affordances for the use of the environment (e.g., by promoting navigation).

Studies on measures of complexity at a meso scale further support the hypothesis that environments which are legible, or easy to “read,” facilitate cognitive skills such as attentional control and navigational skills in older age, as well as promoting usability and engagement in health-related behavior. However, environments need to provide some level of cognitive stimulation to avoid boredom (Rapoport and Kantor, 1967), as shown by the findings that exposure to environments with high fascination and visual richness enhances environmental preference (Kaplan et al., 1989), in turn positively associated with improved selective attention and visual search (Berto et al., 2008, 2010). It is to note that Kaplan et al. (1989) suggested complexity (a measure of visual richness of a scene) and legibility (indicating how easy an environment can be read) as two distinct environmental qualities predicting judgments of preference and perceived restorativeness of environments. This conceptualization seems to contradict our suggestion that legibility could be a potential measure of environmental complexity based on the discussed studies on wayfinding, but we need to distinguish between different levels of operationalization of complexity considering also the role of coherence, another predictor of environmental preference which measures the level of order and organization of an environmental scene (Kaplan et al., 1989). Environments with low legibility are intuitively less coherent, and therefore more complex for perception and cognition, but not necessarily poor in terms of richness of stimulation (or complexity according to Kaplan). On the other hand, an environment can be rich in terms of variation of elements, but still legible and coherent, as in the case of nature. Therefore, both legibility and information-richness inform on the amount of perceptual stimulation received from the environment, and a balance between these two qualities could be a key indicator of cognitively optimal environments.

Lastly, at a macro scale, while exposure to natural (and less complex) settings has the potential to enhance voluntary attention both in young and older samples, and positively impact environmental preference and perceived usability, studies suggest that environments with different levels of structural complexity (e.g., rural vs. urban) can offer different types/levels of stimulation for cognitive health, supporting the role of micro and meso level environmental measures of complexity in influencing cognitive performance both directly and indirectly.

The discussed evidence suggests that environmental complexity can be a key contributor to design living contexts which support and stimulate cognitive health in older age. However, what determines an optimally stimulating environment for older people remains to be established, although the existing measures of complexity support the hypothesis that factors which on one hand facilitate action, and on the other hand stimulate interest could contribute to an optimal level of environmental complexity. This hypothesis should be tested in the context of cognitive aging. Based on the discussed studies, specific suggestions for future research emerge.

Firstly, the most suitable environmental measures to quantify an optimal level of environmental complexity for cognitive performance need to be identified by empirical work. Future experimental studies could manipulate the discussed measures both cross-sectionally to identify correlations with cognitive performance, and longitudinally to highlight causal effects.

The relations between different measures of complexity at different environmental scales should be explored, in terms of understanding whether complexity at a micro scale (e.g., perceptual load) is correlated with complexity at a meso scale (e.g., neighborhood legibility), or whether cognitive abilities engaged at different scales are correlated (e.g., visual search in a cluttered scene and visual search in spatial navigation), or whether the cognitive load required at different scales is associated with preference and, possibly, lifestyle (in terms of use of the environment). Therefore, an analysis of the lived environment could consider, for example, the level of perceptual complexity and restorativeness of specific scenes in the local surroundings (Berto, 2014), the network complexity of the main paths connecting the individual with focal points such as shops, amenities, or parks (Joseph and Zimring, 2007; Slone et al., 2015), as well as the quality of these paths in terms of attentional load and more broadly in terms of aesthetic appeal and perceived usability. This kind of empirical work could then inform both on the mechanisms behind the relationship between environmental complexity, cognition, usability, and preference, and on which environmental characteristics can be modified to make the lived environment more optimal for the aging individual.

Importantly, although many studies on environmental complexity have focused on the visual domain, environments offer multisensory experiences which may impact cognitive processing as well as affective responses and behavior (Brambilla and Maffei, 2006; Wais and Gazzaley, 2011; Emfield and Neider, 2013; Marin and Leder, 2013), and because the processing of information from different sensory modalities changes with age, showing for example a more facilitating effect on attentional performance of multisensory stimuli (Laurienti et al., 2006; Setti et al., 2011), future studies should take into account multiple sensory domains when studying the interaction of older people with their environment.

Both objective and subjective measures of complexity should be tested to identify potential inconsistencies and to attempt a comprehensive operationalization. Long and Baran (2012), for example, found significant correlations between objective intelligibility and perceived legibility of neighborhoods. Moreover, Kim et al. (2014) highlighted the importance of using both objective and subjective measures of the built environment to identify environmental influences on human behavior at multiple environmental scales. The development of surveys and questionnaires could help to assess both objective and subjective environmental factors for cognition, as for example done for the assessment of the pedestrian environment (Clifton et al., 2007), for identifying qualities of residential environments for aging well (Dunstan et al., 2005; World Health Organization, 2007; Burton et al., 2011), or for ratings of preference (Hartig et al., 1997; Laumann et al., 2001).

Lastly, other potential factors should be included in this investigation. For example, the role of coherence (Kaplan et al., 1989) in modulating the relationship between the legibility and the richness of information of an environment should be taken into account when looking at urban design. In addition, familiarity has been shown to influence wayfinding skills (Klencklen et al., 2012; Slone et al., 2015) as well as preference (Berto, 2007), and experience improves driving performance even in complex environments (Underwood et al., 2003; Patten et al., 2006; Underwood, 2007).

The purpose of this work was to provide evidence from the literature that environmental complexity serves as a unifying concept for the multiple environmental influences on cognition, and for studying healthy aging in place from a cognitive perspective, in line with the existing literature on environmental influences on behavior and health (Clarke and George, 2005; Brownson et al., 2009; Beard and Petitot, 2010; Renalds et al., 2010; Carlson et al., 2012; Kerr et al., 2012). The evidence of associations between environmental complexity and cognitive aging is currently fragmentary or inferred from studies on young populations, therefore this targeted review aimed to provide some insights for future research on a topic which is of increasing relevance given global demographic changes (World Health Organization, 2007).

The literature on aging in place (Black, 2008; Mynatt et al., 2000; Wiles et al., 2012) points out the importance of developing effective forms of environmental support which enhance usability, for example through technology (Mynatt et al., 2000; Rantz et al., 2008). Importantly, environmental support needs to be addressed not only in terms of what can be afforded by individual with impairments such as poor vision or hearing, but also in terms of how everyday cognition can be optimized in relation to the environment, an aspect explored, for example, in research on human-computer interaction (Preece et al., 1994; Hollan et al., 2000; Zander and Kothe, 2011; Preece et al., 2015). Understanding cognitive aging in place is a current priority given the increasing need for supportive and enabling environments for aging individuals (World Health Organization, 2007). We argue that studying complexity will advance the knowledge on the factors which make the built environment optimally stimulating for cognition, usable and pleasant, and a first step in this direction is to consider different measures of complexity and their relationships at micro, meso and macro environmental scales. Complementarily, it is crucial to develop instruments to capture how the individual perceives the cognitive load when interacting with the environment and what strategies are adopted to minimize it, for example in case of physical limitations. These instruments should take into account objective measures and the subjective experience of the lived environment.

The proposed framework hopes to stimulate interdisciplinary research on perception, cognition, subjective preference, and usability to better understand environmental influences on cognition, especially in relation to aging, and therefore to inform urban design and planning on strategies to make environments cognitively friendly for older people, where with “friendly” we intend environments which are facilitating but at the same time optimally stimulating.

## Author Contributions

MC and AS conceived the work, drafted and revised the manuscript, gave final approval of the version to be published, and agree to be accountable for all aspects of the work in ensuring that questions related to the accuracy or integrity of any part of the work are appropriately investigated and resolved.

## Conflict of Interest Statement

The authors declare that the research was conducted in the absence of any commercial or financial relationships that could be construed as a potential conflict of interest.
